# Coexistent Breast Cancer and Essential Thrombocythemia: How We Addressed the Therapeutic Challenges

**DOI:** 10.1155/2018/2080185

**Published:** 2018-08-12

**Authors:** Karan Seegobin, Bharatsinh Gharia, Satish Maharaj, Lara Zuberi

**Affiliations:** ^1^Department of Internal Medicine, University of Florida College of Medicine, Jacksonville, FL 32209, USA; ^2^Department of Hematology and Oncology, University of Florida College of Medicine, Jacksonville, FL 32209, USA

## Abstract

Essential thrombocythemia (ET) occurring with breast cancer is uncommon; the therapeutic approach varies and poses a challenge. A 65-year-old female presented to us after being diagnosed with hormone positive, HER2-negative infiltrating ductal carcinoma. She had a platelet count of 600 thou/cu mm. Her JAK2 mutation was positive. Bone marrow biopsy showed increased megakaryocytes. She was diagnosed with ET in the setting of breast cancer. She underwent breast conservation surgery after which aspirin was resumed. Anticipating thrombocytopenia during chemotherapy and given the absence of data combining hydroxyurea with standard chemotherapy used for breast cancer, we felt it prudent to delay cytoreductive therapy for her ET until after completion of breast cancer treatment. Her average platelet count during chemotherapy was 480 thou/cu mm with the lowest being 377 thou/cu mm. Her platelet count remained at goal between 300 and 350 thou/cu mm after four months of hydroxyurea.

## 1. Background

Essential thrombocythemia (ET) is a myeloproliferative neoplasm (MPN) [1]. Patients with this condition have an increased risk to develop a second cancer including both haematological [2] and nonhaematological [2, 3] risks. However, breast cancer in the MPN population is not increased in comparison with the general population [4]. Physicians are faced with several therapeutic challenges when ET coexists with another tumour, in particular solid tumours. These should be managed on an individual basis. Physicians should take into consideration the risk of thrombosis, characteristics of the cancer, and the therapeutic approach for the tumour whether surgical, chemotherapy, or both.

## 2. Case

A 65-year-old female presented with a breast lump, diagnosed to be oestrogen and progesterone receptor positive, HER2 negative, and T1cN1bM0 moderately differentiated infiltrating ductal carcinoma. She had a platelet count of 600 thou/cu mm. There was no history of thrombotic or bleeding episodes.

Further testing showed that JAK2 mutation was positive and t(9;22) mutation was negative. Her bone marrow biopsy showed increased megakaryocytes. Her other lab workup was unremarkable including iron panel and liver function tests. She had no splenomegaly on ultrasound. She was diagnosed with ET in the setting of breast cancer.

Aspirin was commenced but held seven days prior to her breast conservation surgery, restarted postoperatively and continued thereafter. Postoperative thromboprophylaxis with low-molecular weight heparin (LMWH) was continued until the patient was fully ambulatory. Aspirin was restarted on day 7 after the surgery. Anticipating thrombocytopenia during chemotherapy, and given the absence of data combining hydroxyurea with standard chemotherapy used for breast cancer (in this case docetaxel and cyclophosphamide), we felt it prudent to delay cytoreductive therapy for her ET until after completion of breast cancer treatment. Her indication for cytoreductive therapy was >60 years. She was treated with adjuvant docetaxel and cyclophosphamide and continued on aspirin 81 mg for the entire duration of her chemotherapy. She tolerated the 6 cycles of chemotherapy well.

Following the completion of her chemotherapy, she was started on letrozole and radiotherapy with the aim to continue the letrozole for 5 years. Hydroxyurea (500 mg) was also started and titrated to a goal to 400–450 thou/cu mm platelets. Zoledronic acid was started for osteoporosis prevention.

Her average platelet count during chemotherapy was 480 thou/cu mm with the lowest being 377 thou/cu mm (Table 1). Her platelet count remained at goal between 300 and 350 thou/cu mm after four months of hydroxyurea (Table 2). Throughout her treatment, there were no bleeding or thrombotic complications. After one year on letrozole, hydroxyurea, and aspirin, the patient was doing well without complications with platelet counts at goal.

## 3. Discussion

ET is a myeloproliferative neoplasm and is unique among them because of its natural history, which is compatible with a normal lifespan [1]. It is diagnosed by having platelets more than 450 thou/cu mm in the absence of reactive cause, absence of iron deficiency, presence of JAK2 V617F assay, and haemoglobin less than 14 g/dL in women in the absence of splenomegaly [1, 5]. It is a diagnosis of exclusion, representing clonal or autonomous thrombocytosis not classifiable as any of the following diseases: polycythaemia vera, primary myelofibrosis, chronic myeloid leukaemia, or myelodysplastic syndrome [1]. By this criterion, our patient was diagnosed. Other mutations associated with ET are CALR and MPL which carry prognostic significance [6].

Patients with ET have an increased risk to develop a second cancer, with both haematological and nonhaematological cancers being reported [2].

All patients are managed with aspirin provided there exists no contraindications [7]. It has been shown to reduce the risk of arterial and venous thrombosis in low-risk patients [8]. The indications for cytoreductive therapy include those with a history of thrombosis or age greater than 60 years [7]. The degree of thrombocytosis has not been shown to be a reliable indicator of thrombotic risk [7]. Patients with JAK 2 positivity has been shown to have a greater chance of arterial and venous thrombosis [7]. Hydroxyurea is the only cytoreductive agent proven to reduce thrombotic events in a randomized controlled trial and is the first-line cytoreductive agent [9].

The goal of cytoreductive therapy is to resolve symptoms and normalize the platelet count [7].

Although perioperative thrombotic and bleeding complications appear increased in ET patients, it is not clear whether this can be ameliorated by therapeutic intervention [10]. The management is difficult because there are no definite guidelines available in the literature discussing this issue [11].

Determining the method of cytoreduction is a challenge, as it is important to achieve a balance between such dangerous postoperative complications as vascular thrombosis and excessive haemorrhage [11]. In one report, hydroxyurea was the drug of choice to achieve effective cytoreduction in patients with symptoms of ET—who are intended to undergo cardiac surgery [11]. For patients receiving cytoreductive therapy who are undergoing elective surgery, the blood count is optimized preoperatively, and interruptions in therapy are kept to a minimum [7]. Postoperative thromboprophylaxis is recommended according to usual guidelines for the specific procedure [7]. For patients not receiving cytoreductive treatment, temporary therapy is considered on a case-by-case basis, after assessment of the patient's thrombotic risk profile, degree of thrombocytosis, and the nature of the surgery [7].

Other malignancies such as tongue cancer, gastric cancer, and colon cancer have been reported in the setting of ET [12–14]. ET is likely a coincidental finding in this breast cancer case.

Thrombocytopenia is a well-known side effect of chemotherapy [15, 16]. Considering this, we completed the course of chemotherapy prior to starting hydroxyurea for the patient's essential thrombocythemia. As expected, our patient's platelets counts had a downward trend during her course of chemotherapy.

One study conducted on low-risk ET patients showed that antiplatelet therapy reduces the incidence of venous thrombosis in JAK2-mutated patients and the rate of arterial thrombosis in those with cardiovascular risk factors [8]. Aspirin was continued during her chemotherapy treatment, and the patient was monitored closely for bleeding complications. Hydroxyurea was not started during chemotherapy as she did not have a history of thrombotic complications, and we expected her platelet count to drop during the chemotherapy treatment. She did not suffer from thrombotic or bleeding complications during her chemotherapy treatment. A decision to institute cytoreductive therapy should be based on an individual basis.

Survival of ET patients does not substantially differ from that of the general population [17]. On the contrary, the outlook for women with breast cancer varies by the stage of cancer [18]. In general, the survival rates are higher for women with earlier stage cancers [18]. The outlook for each woman is specific to her circumstances [18]. The 5-year relative survival rate for women with stage 0 or stage I breast cancer is close to 100% [18]. For women with stage II breast cancer, the 5-year relative survival rate is about 93% [18]. ET and breast cancer are two malignant disorders with favourable outcomes, and therefore, treatment for one should not preclude treatment for the other.

It is important to recall that letrozole and other aromatase inhibitors can increase the risk of venous thromboembolism (VTE) [19]. Furthermore, in our patient, ET increases her risk for VTE, thus the importance of hydroxyurea to keep platelets at goal cannot be overstated. We have not found any cases of adverse drug interactions between letrozole and hydroxyurea. Furthermore, our patient has been doing well without any adverse effects of such drug combinations. Solid tumours and chemotherapy both increase risk of blood clots [20, 21]. VTE is one of the causes of mortality reported in patients on chemotherapy, though this is less likely in an early stage cancer with low disease burden [21]. In our patient, the risk of VTE from her early stage breast cancer and chemotherapy would likely compound the risk from the underlying myeloproliferative neoplasm, so it is likely essential to counsel these patients on reducing risk by optimizing mobility. Additionally, antithrombotic prophylaxis should be considered during chemotherapy. While it is not routinely recommended in the outpatient setting, it should be considered for high risk patients [22]. The current guidelines indicate that low-molecular weight heparin is the first choice [22]. In our case, we continued postoperative VTE prophylaxis until the patient was fully ambulant; however, every case should be reviewed in a holistic way with consideration given to extending VTE prophylaxis if required. In patients undergoing major abdominal or pelvic surgery with high-risk features, VTE prophylaxis is recommended up to 4 weeks and in other cases, extended up to 6 months [22]. These patients should be periodically reassessed at each follow-up visit to review their risk for VTE [22].

## 4. Conclusion

- Physicians are faced with several therapeutic challenges when ET coexists with solid tumours.
- These should be managed on an individual basis.
- One should take into consideration the risk of thrombosis and the therapeutic approach for solid tumours in the management of these patients.
- Extended VTE prophylaxis during chemotherapy may be needed in select cases.
- ET and breast cancer both have favourable outcomes, and therefore, treatment for one should not preclude treatment for the other.

## Figures and Tables

**Table 1:** Complete blood count (CBC) before, during, and after chemotherapy.

CBC	Before chemotherapy	After 4 cycles of chemotherapy	After 5 cycles of chemotherapy	After 6 cycles of chemotherapy	Two months after completion of chemotherapy
White cell count (×10^3^/*μ*L)	6	3	3	6	7
Haemoglobin (g/dL)	13	12	12	13	12
Platelet (thou/cu mm)	631	592	497	377	457

**Table 2:** Complete blood count after hydroxyurea.

CBC	Two months after starting hydroxyurea	Four months after starting hydroxyurea
White cell count (×10^3^/*μ*L)	7	6
Haemoglobin (g/dL)	12	12
Platelet (thou/cu mm)	326	325
